# Predicting the Deviation from the Standard Study Period and Dropout Intentions Through Depression Severity and Social Integration Among University Students in Germany: A Longitudinal Analysis

**DOI:** 10.3390/ijerph22050667

**Published:** 2025-04-24

**Authors:** Antje Römhild, Alfons Hollederer

**Affiliations:** Department of Social Work and Social Welfare, The Faculty of Human Sciences (FB 01), University of Kassel, 34127 Kassel, Germany; alfons.hollederer@uni-kassel.de

**Keywords:** university students, academic performance, mental health status, mental health services, Patient Health Questionnaire Depression (PHQ)

## Abstract

Mental health problems among university students have become a growing concern for higher education institutions. Students experiencing mental health issues, with depression being the most prevalent disorder, tend to take longer to graduate and have a higher risk of dropping out of university. This study examines the predictive values of depression severity (Patient Health Questionnaire Depression—PHQ-9), use of psychosocial counseling, and social integration on the deviation from the standard study period and dropout intentions. A total of 3300 students at the University of Kassel, Germany were surveyed at baseline in March 2022; 1744 students provided an email address and gave permission to contact them individually for the follow-up survey in March 2023. After eliminating dropouts and questionnaires with a lot of missing values, the final sample consisted of 500 students who participated at both time points. Longitudinal data were used for descriptive, correlational, and multiple linear regression analyses. Multiple linear regression analyses revealed a significant adverse predictive value of the PHQ-9 (β = −0.082; *p* < 0.05) on the deviation from the standard study period. The analyses found significant positive predictive values of the PHQ-9 (β = 0.190; *p* < 0.001) and examination grades (β = 0.108, *p* < 0.05) on dropout intentions. Furthermore, this study could not confirm significant predictive values of difficulties with interaction with fellow students and lecturers on dropout intentions. The results highlight the role of health promotion, psychosocial counseling, and social networks for students with depressive symptoms. Concluding, a networked approach at universities involving students, lecturers, counseling services, and health management is recommended.

## 1. Introduction

A mental health crisis has reached university campuses worldwide (USA: [1]; Europe: [2,3]; UK: [4]; Africa: [5]; Asia: [6,7]; Australia: [8]). According to several studies, the number of students experiencing mental health problems is high, with depression being the most prevalent mental disorder among tertiary students [9,10]. The “WHO World Mental Health International College Student Project (WHM-ICS)”, a cross-national study of *n* = 13,984 in eight countries, indicates that between October 2014 and February 2017, at least one-third of students reported experiencing mental health disorders. Furthermore, the 12-month prevalence of major depressive disorder (MDD) was approximately 18.5% [11]. A subsequent study by the WMH-ICS initiative, using data from 2017 to 2023 and including *n* = 72,288 university students in 18 countries, found a 12-month prevalence rate for mental health disorders of 57.4% and for MDD of 20.5% [12]. The “Healthy Minds Study”, a comprehensive investigation that gathered data from 373 colleges in the USA, revealed that three-quarters of students participating in the survey between 2020 and 2021 exhibited psychological distress at moderate to severe levels. Furthermore, the investigation revealed that 60% of the students met the criteria for one or more mental health problems [13]. There is evidence that this deterioration of mental health among higher education students is a side effect of the pandemic developments of recent years: The COVID-19 pandemic that began in 2020 was associated with several educational, social, and financial challenges for university students worldwide. Due to infection control measures, such as lockdowns and quarantine, many students lost their jobs and financial support from their families became uncertain. This has exacerbated the financial insecurity of studying, while students have had to cope with limited home learning resources [14]. As a result of this situation, the prevalence of distress, loneliness, depression, and anxiety has increased significantly among university students worldwide [7,15,16,17]. A meta-analysis by Deng et al. [18] of *n* = 1,441,828 found that the prevalence of depressive symptoms among university students during the pandemic reached 34%.

Another aspect that explains the high prevalence of mental health problems is that the commencement of academic studies goes hand in hand with the process of detachment from the family environment and the transition to an independent life, which is closely linked to identity development [19]. During this period, university students face more substantial changes and novel challenges in comparison to their working counterparts. They must adapt to a new organizational structure for their studies and face increased workload pressures. Concurrently, students encounter financial insecurities and confront dual burdens, encompassing both academic demands and supplementary employment. This can be associated with high levels of psychological distress and, concomitantly, the loss of social networks. Consequently, many college students encounter the initial onset of mental health problems or an exacerbation of their symptoms [20,21,22].

### 1.1. Mental Health Problems and Academic and Social Challenges Among University Students in Germany

Even before the COVID-19 pandemic, university students in Germany were increasingly suffering from mental health problems [23]. The pandemic has had a significant impact on students’ well-being, with many students reporting increased anxiety and fear of infection. This has been associated with a further rise in depressive symptoms [24]. According to a recent report on the health of tertiary students by a health insurance company [25], the proportion of students reporting frequent feelings of stress was 44%, and 34% of them experienced depressive symptoms.

Students being affected by depression have been shown to demonstrate elevated levels of academic and social impairment: Depressive symptoms can influence cognitive functioning and attention, potentially resulting in avoidance behaviors and challenges with academic tasks and social interactions [22,26,27]. Research findings suggest that students dealing with mental health concerns encounter more substantial challenges in domains like study organization, workload management, and social interactions compared to their peers [28,29,30].

In particular, interactions during studies have been shown to play a key role in facilitating social integration. However, students with mental health problems report less interaction with peers and faculty compared to students without these problems [28]. Only one in five students reports feeling comfortable discussing their mental health condition with their peers. Similarly, regarding communication with faculty, only about one-third of students report that their lecturers understand their situation [28]. Concurrently, students may be reluctant to disclose mental health problems due to concerns about the potential stigma and negative attitudes that could affect their academic or professional performance [31]. Hence, these students are less capable of benefiting from the experience and expertise of their peers as well as the support of their instructors [32].

Ultimately, the above-mentioned academic and social problems can put students with mental health problems at a disadvantage compared to other students [21,33]. This finding is supported by the “best 3 study-Studying with a Health Impairment” [28] with *n* = 179,908, which showed that this student group was more likely to have interrupted their studies at least once (21.6% vs. 9.0%), to have changed subjects at least once (37.8% vs. 23.6%), or to have considered dropping out (14.8% vs. 4.7%) in comparison to students without health-related study problems [28,34].

To reduce these adverse effects, early identification and treatment of mental health problems is essential [35]. Therefore, the provision of low-threshold psychosocial counseling by institutions of higher education and student services is intended to furnish guidance on coping with academic challenges and social difficulties [15]. Additionally, it facilitates referrals to supplementary psychotherapeutic counseling services [36,37]. Previous studies have indicated a notable increase in demand for mental health services on university campuses in recent years [4,13]. However, research from Germany has documented that students with mental health issues are less likely to utilize existing institutional support when compared to students with physical or sensory impairments [29]. These students often do not seek support because they do not define themselves as eligible, do not want to be treated preferentially, or fear disadvantages in their studies and careers [29,38]. According to a review by Hartrey et al. [39], the reasons for this may include a lack of mental health literacy and awareness regarding the support services offered by the university.

### 1.2. Theoretical Background: Research on Academic Performance and Dropout Intentions of University Students

The extant literature pertaining to academic performance and dropout in higher education is multidimensional, incorporating a wide array of sociological, psychological, and economic explanatory models [40,41,42]. Recent research has focused on either the social aspect of student integration or on structural factors that predict academic success and students’ dropout intentions [41]. The predominant approach to this issue is Tinto’s Model of Institutional Action [43,44]. The model integrates individual characteristics (gender, age, class, health), the external setting (e.g., family, work, community), and structural factors (e.g., financial circumstances, faculty, staff). However, the subject of controversial debate is to what extent the model, developed in the context of the US higher education system, can be transferred to other higher education contexts [45].

According to the European Commission, academic performance in higher education encompasses a range of student achievements. In addition to the grades obtained in examinations, these achievements may include the completion of studies, the time-to-degree, and student retention or dropout from studies [46]. Previous research defines dropout from university as the final decision to leave higher education [45]. However, the measurement of dropout is often carried out retrospectively using exmatriculation surveys, which are associated with high non-response rates and recall errors [47]. According to Bäulke et al. [42], a dropout decision is preceded by a longer-lasting decision-making process that includes the consideration of dropout intentions, the search for reasons, and the identification of further options after withdrawal. Documenting dropout intentions, therefore, serves to address the methodological challenge of quantifying actual dropouts from the higher education system.

### 1.3. Objectives and Hypotheses

The relationship between students’ mental health, academic performance, and dropout intentions, as well as the impact of psychosocial counseling and social integration, has been studied sporadically but has not yet been sufficiently researched [8,48,49,50]. However, understanding what inhibits academic success and what promotes student retention is essential for student support services, student health management, and for higher education institutions. This study aims to close this research gap and focuses on students with depressive symptoms:

International research has shown direct or mediating adverse effects of psychological distress [51] and students’ self-reported depressive symptoms on academic performance in terms of grade point average (GPA) [26,27,52,53]. With regard to study duration, several studies indicate that students with health-related study difficulties generally take longer than average to complete their education [54,55,56,57], and that their completed course load is significantly lower than required by study regulations [58]. However, there is a paucity of research regarding the effects of depressive symptoms on the deviation from the standard study period. The following hypothesis is derived to examine this association:

**Hypothesis** **1** **(H1):** *The higher the level of students’ depression, the higher the deviation from the standard study period*.

Psychotherapeutic counseling for students at universities in Germany has demonstrated efficacy in practice, as evidenced by improvements regarding the severity of psychological distress and the increase in study satisfaction among those who have utilized these services [21,59]. However, there is a lack of research on the relationship between psychosocial counseling and study duration. Therefore, it would be beneficial to examine the potential impact of psychosocial counseling on the deviation from the standard study period:

**Hypothesis** **2** **(H2):** *The use of psychosocial counseling (psychological/social advice) will decrease the deviation from the standard study period for users compared to students with equivalent severity of depression who did not use psychosocial counseling.*

Students with mental health problems report to face several challenges and experience low social support in the university setting [31,33,39]. This results in being at a disadvantage compared to other students and, ultimately, a higher risk of dropping out of higher education [8,53]. In the context of German higher education, health problems are among the primary factors contributing to the dropout decision in 10% of students; for 4%, it is even the decisive reason [45]. However, research examining the relationship between mental health and students’ dropout intentions is comparatively limited and the extant literature is constrained by methodological and scope limitations [8]. A few studies demonstrate that university students treated for mental health problems [8], students with psychic disabilities [60], and students with depressive symptoms [61] exhibited a higher risk of dropping out of studies. The following hypothesis results from this:

**Hypothesis** **3** **(H3):** *The higher the level of depression, the higher the students’ dropout intentions.*

Due to the aforementioned challenges with regard to examinations and the organization of studies, students with mental health issues are more likely to interrupt their studies or change subjects, which leads to prolongation of their studies [28]. While there appears to be no overall difference in grades, students with mental health problems in particular tend to rate their academic performance significantly lower than students without these issues [60]. Tinto’s Model of Institutional Action is predicated on the assumption that academic performance serves as a predictor of dropout from higher education [43], which has been confirmed by several studies [62]. Russmann et al. [60] examined the association between students’ academic performance and dropout intentions and showed that students with better academic performance are better protected against high dropout intentions. This leads to the following hypothesis:

**Hypothesis** **4** **(H4):** *The worse the examination grades or the higher the deviation from the standard study period, the higher the students’ dropout intentions.*

Recent research has validated the role of social integration as a predictor [63] and study climate as a mediator [64] in predicting dropout intentions. A few studies underlined the importance of social support from peers and faculty to improve mental health [65,66,67]. These interactions have been shown to mitigate students’ academic disadvantages [32] and prevent the risks of dropping out of university [60,61]. Consequently, social interactions may emerge as a significant resource for health and the ability to work and study [68], potentially mitigating dropout risks.

**Hypothesis** **5** **(H5):** *The lower the students rate their difficulties with social interactions with fellow students and lecturers, the lower their dropout intentions*.

## 2. Materials and Methods

### 2.1. Research Design, Ethical Approval, and Data Collection

The University of Kassel is a medium-sized German state university with a multi-layered competence network of expertise in various subjects such as natural and technical sciences, arts, humanities, and social sciences [69]. In March 2022, 23,699 University of Kassel students who studied in the winter semester 2021/22 were invited to take part in an online survey (“computer assisted web interview”) via Lime Survey. The survey was fully anonymized. Of the total, 1744 students provided us with an email address at the end of the survey and permitted us to contact them individually at a later date. These students were invited via email to participate in the follow-up survey in March 2023. Students received emails with a letter of motivation and an encrypted access link to the email accounts set up by the university upon enrolment. Participation was voluntary. Several reminders were sent during the survey period. Items from the university store were raffled as an incentive. This study and the content of the survey were approved by the University of Kassel’s Ethics Committee (EK-No. 202128, 15 December 2021) and were conducted in accordance with the Declaration of Helsinki [70]. Prior to the survey, a pre-test was conducted by the institute’s staff with 10 participants. Subsequently, existing methodological problems with the questionnaire were revised. The baseline survey sample is described in Hollederer [58] and Hollederer and Dieckmännken [71].

### 2.2. Measures and Instruments

The baseline and follow-up survey questionnaire consisted of modules on sociodemographic information, study situation, health variables, and variables on academic performance and dropout intentions. Variables related to self-reported health and study-related items are valid constructs adopted from recent studies [23,28,30,48].

The selected predictor and outcome variables with their means, standard deviations or proportions, and Cronbach’s α, where applicable, are shown in Table 1:

The subjective assessment of depressive symptoms as a predictor variable was operationalized by the Patient Health Questionnaire Depression (PHQ-9), a battery of items with 9 different statements [72]. Answers to each item were rated on a 4-point Likert scale from 0 = “not at all” to 3 = “nearly every day”. The Patient Health Questionnaire Depression (PHQ-9) is a reliable and valid instrument for measuring the severity of depression with a good internal consistency of Cronbach’s α = 0.86. For analysis, the 9 items (range 0–3) were aggregated to a sum variable with a range from 0 to 27. A PHQ-9 score of ≤10 is the cut-off for major depressive disorder (MDD). In the sample, approximately one-third of the students met the criteria for MDD (T0: 31.2%; T1: 32.6%).

The second pair of predictor variables referred to the use of psychosocial counseling: Participants were asked to respond to items about the use of psychological and social advice [73] offered by the Studierendenwerk (Student Union) Kassel. Answers were rated on a 3-point scale (1 = “I have used”; 2 = “I know, but have not used them”; 3 = “I do not know”). For analysis, two dummy variables were calculated with the values 0 for “not used” and 1 for “used”. In our sample, at T0 less than 10% of the students expressed to have used psychosocial counseling (psychological advice: 8.4%; social advice: 4.6%). These proportions increased at T1 (psychological advice: 13.6%; social advice: 6.5%).

For this analysis, academic performance was measured by self-reported examination grades (range: 1.0 = “very good”; 5.0 = “poor”) and deviation from the standard study period as predictor variables. The latter was calculated from the difference between self-reported standard study period and individual study time. The arithmetic path is defined as follows: “*How many semesters is the standard study period in your current degree program?”- “And in how many semesters do you expect to complete your current degree program, including the examination semester?*”. The range of item values was between −11 and 6, with negative values implying a longer study duration compared to the standard study period and positive values implying a shorter study duration compared to the standard study period (T0: M (SD) = −1.69 (2.23); T1: M (SD) = −2.02 (2.37)).

In addition, two single items based on the 13th German Student Survey [74] were introduced to measure social integration through difficulties with interactions with fellow students and lecturers. Responses were based on a 4-point Likert scale with increasing scores corresponding to stronger difficulties (1 = “none”–4 = “strong”). Approximately half of the students reported several or strong difficulties in interactions with fellow students (M (SD) = 2.51 (0.97)), while only 24.4% reported the same for contact with lecturers (M (SD) = 2.0 (0.83)). There was a decrease in these proportions at T1.

This study introduced the deviation from the standard study period as the first outcome variable (see description above). The second outcome variable, dropout intentions, was operationalized by students’ responses to two statements related to non-fit perception and thoughts of quitting studies completely from the battery of items related to the process of dropping out according to Bäulke et al. [42]. For the analyses, a mean variable with an acceptable Cronbach’s α of 0.75 was calculated from the two 5-point Likert-scale items (T0: M (SD) = 1.74 (0.86); T1: M (SD) = 1.84 (0.99)).

Dahm et al. [63] found that individual life circumstances, and previous educational outcomes predict dropout intentions. Further research showed that women and older students, in particular, felt more burdened by study requirements and exams and were more likely to be stressed by the additional demands of studying, and working while studying [25,28]. In addition, recent research indicates that students in bachelor’s degree programs report more health-related study difficulties than do students in other degree programs [28].

Therefore, grades of the study entrance certificate (range 1.0–5.0) and the financial burden associated with studying using a 5-point scale were introduced as further predictor variables.

Sociodemographic variables (female gender, age) and degree were assessed using standard survey questions (for proportions see Table 2). Further, dummy variables were created for these variables.

### 2.3. Statistical Analyses

Data analyses were performed using IBM SPSS-Statistics version 28 (New York, NY, USA). The research approach used standard descriptive analyses, and confirmatory factor analyses where applicable, depending on the number of items. To identify multicollinearity, pairwise correlation analyses were conducted for all relevant predictor and outcome variables (see Table 3). Since correlations for categorial variables were calculated, non-parametric testing was used. According to Field [75], the rank correlation coefficients should, therefore, not exceed 0.8. In multivariate analyses, ordinary least squares (OLS) linear regression models [76], were tested to determine the effects of the hypothesized predictor variables on the outcomes. Two variables representing the baseline values of the respective outcomes were included in the regression models, because the baseline value of the outcome (T0) is expected to predict the dependent outcome variable (T1) and may therefore be a possible confounder in the model. In order to avoid confounding effects, gender and age—expected to affect outcomes—were controlled for in all procedures. Gender was treated as a dichotomous measure (1 = “female”; 0 = “male”) and female gender was incorporated into the models as a predictor variable (see Table 4 and Table 5). In non-randomized studies, the presence of confounders can be a source of bias in the effects of other independent variables [77]. All analyses were conducted as block inclusion analyses. One-sided *p*-values were used to assess the results according to the a priori scientific hypotheses (H0) and the alternative statistical hypotheses (H1–H5).

## 3. Results

### 3.1. Sample Characteristics

A total of 3330 of the 23,699 enrolled University of Kassel students completed the online survey via Lime Survey in March 2022. This corresponds to an overall response rate of 13.9% at T0. Of the 1744 students who provided their email address, the final sample at T1 consisted of 500 students who were studying at the University of Kassel in the winter semester 22/23, after dropout and missing data reduction. This represents a response rate of 28.7% of students invited to participate in T1. The overall response rate of students from T0 who participated in T1 is 15.2%, which is consistent with the usual student response rate during the COVID-19 pandemic [78].

Sociodemographic characteristics for the baseline survey are shown in Table 2. The majority (75.2%) of students belonged to the 21–30 age group, 12.2% were younger than 21, and 12.6% older than 30 years. The majority of the sample (n = 326; 66.7%) was female, while 33.2% (n = 166) were male respondents. Only 1.4% of the students (n = 7) reported a diverse gender identity; due to this low proportion, diverse identity is not shown in the table. 7.2% of the students (n = 36) reported a foreign nationality. One-third of the respondents (29.6%; n = 148) were studying in their first semester. At baseline, approximately half of the respondents (53.6%; n = 268) were studying in bachelor’s programs, 28.0% (n = 140) in master’s programs, 15.0% (n = 75) in teacher education programs, and 3.4% (n = 17) in other degree programs.

### 3.2. Bivariate Correlation Analysis

Table 3 shows the pairwise correlations between all the predictor variables and the outcome measures. Regarding the direction of the correlations, the PHQ-9 indicator variable showed significant and plausible correlation coefficients with both outcome variables at T1. The scores are consistent with the alternative hypotheses H1 and H3. This means that the severity of depression (PHQ-9) at T0 is negatively correlated with the deviation from the standard study period and positively correlated with dropout intentions at T1. The values range from *r*s = −0.19 (correlation between PHQ-9 and deviation from standard study period [weak negative correlation, statistically significant]) to *r*s = 0.34 (correlation between PHQ-9 and dropout intentions [weak positive correlation, high significance]). Statistically significant and moderate correlations ranging from *r*s = 0.51 to *r*s = 0.66 were found between the baseline (T0) and follow-up (T1) scores of the outcome variables deviation from the standard study period and dropout intentions. For use of counseling services, significant correlations were found with the following outcome measures at T1: Use of psychological advice had significant weak negative correlations with deviation from the standard study period (*r*s = −0.19, *p* < 0.01) and positive correlations with dropout intentions (*r*s = 0.10, *p* < 0.05). Use of social advice also showed significant negative correlations with deviation from the standard study period (*r*s = −0.12 *p* < 0.01). Examination grades had significant correlations with deviation from the standard study period (*r*s = −0.15, *p* < 0.01) and dropout intentions at T1 (*r*s = 0.27 *p* < 0.01). Difficulties in social interaction with fellow students at T0 showed significant negative correlations with deviation from the standard study period (*r*s = −0.14, *p* < 0.01) and dropout intentions at T1 (*r*s = 0.28 *p* < 0.01) while difficulties with interaction with lecturers had weak significant negative correlations with deviation from the standard study period (*r*s = −0.13, *p* < 0.01) and weak significant positive correlations with dropout intentions at T1 (*r*s = 0.20, *p* < 0.05). Furthermore, there were also weak significant correlations between grade of study entrance certificate, financial burden, age, and deviation from the standard study period at T1 and between degree, grade of study entrance certificate, financial burden, and dropout intentions at T1. The strongest correlations were identified between the T0 scores on the PHQ-9 (*r*s = 0.34; *p* < 0.01) with dropout intentions at T1, and between interaction with fellow students and dropout intentions (*r*s = 0.28, *p* < 0.01) as previously discussed. Finally, there were no strong correlations between the predictor variables with Spearman rank coefficients greater than 0.8 according to Field [75], so it can be assumed that there is no multicollinearity. 

### 3.3. Multivariate Linear Regression Analyses

Multivariate linear regression analyses were conducted to test for causal associations between the predictor variables and the outcome variables of deviation from the standard study period and dropout intentions. The standardized regression coefficients (β), the coefficients of determination (R^2^), and the adjusted coefficients of determination (R^2^adj.) are presented in Table 4 and Table 5. Prior to each formal analysis, the assumptions of the linear regression models (i.e., linearity, absence of collinearity and heteroscedasticity, normality of residuals) were checked for. The models had no autocorrelation, as the value of the Durbin–Watson statistic is between 1.88 and 2.01. In our models, the lowest value for tolerance is 0.75. It can thus be concluded that there is no multicollinearity between the predictors. The residuals are also normally distributed according to this test.

**Hypothesis** **1** **(H1):** *The higher the level of students’ depression, the higher the deviation from the standard study period.*

The third regression model (M3 in Table 4) shows that students with a 1 scale point higher level of depression tend to have a 0.082 semesters higher deviation of individual study time from the standard study period than students with lower scores on the PHQ-9 scale (β = −0.082; *p* < 0.001), even when controlled for the outcome of deviation from the standard study period at T0 (baseline outcome), female gender, and age. Additionally, female gender (β = 0.070; *p* < 0.05) was found as a significant predictor of deviation from the standard study period, while age (*p* = 0.310) did not significantly predict the outcome. The (H1) hypothesis, that a higher severity of depression is associated with higher deviation from the standard study period, can therefore be confirmed.

**Hypothesis** **2** **(H2):** *The use of psychosocial counseling (psychological/social advice) will decrease the deviation of individual study time from the standard study period for users compared to students with equivalent severity of depression who did not use psychosocial counseling.*

When two different variables of psychosocial counseling were added to the model (M4 in Table 4), the standardized regression coefficient of depression severity decreased from −0.082 to −0.072 scale points (*p* < 0.001). In addition, the association with female gender remained significant (β = 0.069; *p* < 0.05). At the same time, it was found that the use of psychological advice (*p* = 0.147) and social advice (*p* = 0.371), did not significantly predict the deviation from the standard study period in M4. Consequently, Hypothesis 2 (H2) must be rejected.

In the final model M5 in Table 4, additionally, after inspection of pairwise correlations with the outcome of deviation from the standard study period, the variables grade of entrance certificate and financial burden were included in the model. After adding covariates, the PHQ-9 variable was no longer a significant predictor of the outcome. The covariates did not significantly predict the deviation from the standard study period, and they did not account for a significant increase in the explained variance of the outcome. One of the main results of this first OLS linear regression analysis is that the PHQ-9 variable is identified as the strongest predictor variable of deviation from the standard study period. The model M4 was statistically significant and explained 50.7% of the variance in the outcome. With an R^2^ of 0.514 and an adjusted R^2^ of 0.507 (F(6,476) = 83.74; *p* < 0.001), the variance explained was strong according to Cohen [79].

**Hypothesis** **3** **(H3):** *The higher the level of the students’ depression, the higher the students’ dropout intentions.*

Table 5 shows the results for the linear regression models (M1 to M6) for the outcome of dropout intentions at T1. As illustrated by the third model (M3 in Table 5), there is a weak but statistically significant relationship between the severity of depressive symptoms and dropout intentions (β = 0.204; *p* < 0.001). Furthermore, the control variables female gender (*p* = 0.873) and age (*p* = 0.935) did not significantly predict the outcome variable. In line with H3, it was found that when students’ level of depression increases by 1 scale point, their intentions to drop out of university also increase by 0.204 scale points. This means that the risk of dropping out of university is higher for students with higher scores on PHQ-9.

**Hypothesis** **4** **(H4):** *The lower the examination grades and the longer the study duration, the higher the students’ dropout intentions*.

When the two variables examination grades (T0) and deviation from the standard study period (T0), which represent academic performance, were included in the model, the significant effect of the PHQ-9 on dropout intentions decreased to 0.190 scale points (*p* < 0.001), while female gender (*p* = 0.890) and age (*p* = 0.875) did not predict the outcome. However, Model 4 shows that examination grades is a significant positive predictor of dropout intentions (β = 0.108; *p* < 0.05), while the deviation from the standard study period did not significantly predict dropout intentions (*p* = 0.115). In partial support of Hypothesis 4, students with an exam performance one grade better (range 1–5, with 1 being the best grade) show 0.108 scale points lower levels of dropout intentions (F(6,325) = 25.567; *p* < 0.001).

**Hypothesis** **5** **(H5):** *The lower students rate their difficulties with social interactions with fellow students and lecturers, the lower their dropout intentions.*

Furthermore, after adding the social integration measures to the model (M5 in Table 5), the standardized regression coefficients of depression severity (β = 0.169; *p* < 0.001) and examination grades (β = 0.106; *p* < 0.05) were slightly reduced but the variables remained significant predictors of dropout intentions. However, deviation from the standard study period did not significantly predict the outcome (*p* = 0.212). The association of difficulties with interaction with fellow students (β = 0.099; *p* < 0.051) and dropout intentions was positive but not significant and the coefficient for the difficulties with interaction with lecturers was negative and not significant (β = −0.021; *p* = 0.663). Hypothesis 5 (H5), which indicated that difficulties in interaction with fellow students and lecturers are predictors of dropout intentions, can therefore not be confirmed. The variables degree and financial burden in Model 6 did not result in a significant improvement in the explained variance of dropout intentions. Therefore, the R^2^ for the best fitting model M5 was 0.329 (R^2^adj. = 0.312); (F(8,323) = 19.76; *p* < 0.001), indicating a moderate goodness of fit according to Cohen [79]. It can therefore be concluded that two of the hypothesized variables are significant predictors of dropout intentions.

## 4. Discussion

The present study contributes novel evidence to the research on mental health and academic performance of university students. To this end, longitudinal data from 500 university students who participated in a student survey at the University of Kassel in 2022 and 2023 was employed in the multivariate regression analyses. This approach enabled the determination of predictive relationships from one year to the next between depression severity, academic performance, and potential protective factors. In the field of student mental health research, the incorporation of longitudinal data represents a significant advancement in relation to the predominantly cross-sectional studies that have been carried out in the past. To the best of our knowledge, this is the first longitudinal study conducted in Germany that attempts to combine and answer these specific questions.

The descriptive results show that approximately one-third of the students at the University of Kassel met criteria of elevated levels of depression. This phenomenon is indicative of a trend that has been further exacerbated by the effects of the recent (COVID-19) pandemic [18] and which has been previously documented in several of international [4,13] and German studies [21,23,58].

The present longitudinal analysis demonstrates that depression severity has a substantial adverse impact on the deviation from the standard study period, resulting in prolonged study periods. This finding is consistent with the conclusions of studies by Plasa [57] and Hollederer [58], which confirmed associations between health difficulties, study duration, and completed study load. One potential explanation is that depressive symptoms—which lead to a lack of motivation and difficulty concentrating—have been shown to have a negative impact on academic performance [26,27]. On the other hand, students with mental health problems encounter significant challenges in organizing their studies and exams, as well as managing their workload. Concurrently, these students have been found to interrupt their study programs or change their academic disciplines more frequently than students without these problems [28,29].

Despite the documented efficacy of psychological counseling in reducing psychological distress and increasing satisfaction among university students [36,59], this study did not confirm that the use of psychosocial counseling (psychological/social advice) would reduce the deviation from the standard study period for users compared to other students with the same level of depressive symptoms. The fact that only a small percentage of the sample received psychosocial counseling is the most likely explanation for this negative finding. Since the signs of the standardized regression coefficients for counseling use are negative, the question arises whether the use of counseling is associated with longer study times. It is important to note that the implementation of an effective counseling process to address study challenges and augment students’ resources requires a significant time investment, which may necessitate an extension of their study time [36,37].

Furthermore, this study found that higher levels of depression were associated with higher dropout intentions. These findings are consistent with those of several studies, which found that students with mental health problems and depression are at higher risk of dropping out from university [8,53,60,61]. This result may be associated with non-disclosure and stigma, which are frequently associated with mental health problems, such as depression. This phenomenon can potentially lead to a decline in academic performance, which, in turn, may ultimately increase the risk of early dropouts from higher education [8,53].

Examination grades were found to positively predict dropout intentions. This finding aligns with the conclusions of Rußmann et al. [60], who demonstrated that satisfaction with academic achievements exerts a substantial protective effect on the intention to drop out. It has been observed that students dealing with mental health issues are more prone to interrupt their studies due to illness or switch subjects, which results in extended study periods [28]. However, the present study found that the deviation from the standard study period did not significantly predict the intention of university dropout.

In contrast to several studies that underlined the importance of social support from peers and faculty to improve mental health [65,66,67] and the role of social integration as a predictor [63] and protective factor [60,61] of dropout intentions, the present study could not confirm difficulties with interactions with fellow students and lecturers as predictors of dropout intentions. A plausible explanation for the non-significant results is that we only used single items, whereas other studies used more valid measures. For this reason, the comparability of the results is limited. Nonetheless, interactions during studies play an important role in facilitating social integration. However, descriptive results showed that interaction with peers resulted in more difficulties than those with lecturers and supported the results of other studies [28,31].

### 4.1. Limitations and Strengths

One of the methodological strengths of this study is that it is a complete survey of students at the University of Kassel. This survey methodology avoids typical sources of error such as sampling or random error. By knowing the population, influencing factors such as gender or age can be identified in the response rates and controlled for in the multivariate analyses. Including these control variables strengthens the validity and reliability of the results [71].

The results have a number of potential limitations: First, the survey instrument used, a computer-assisted web interview, has limitations: it was used exclusively to collect self-reports from students on their subjective perceived health without the presence of medical diagnoses. In addition, all information on academic performance and dropout intentions is based on self-assessment only. Therefore, it must be acknowledged that social desirability may play a role in response behavior and response bias could not be entirely excluded.

Participation in the survey was voluntary for students, and response to the baseline and follow-up surveys was not high under pandemic conditions [58]. The chosen sampling method reduced the response rate and introduced a self-selection bias in the final longitudinal sample. Given the collection of sensitive data concerning health and academic performance, a certain rate of attrition could not be ruled out.

The main limitation of the linear regression analyses is that they could not fully explain the observed variance in the outcomes of deviation from the standard study period and dropout intentions. The effects of other mental health assessments and other unknown variables may increase the explained variance of the results.

The findings are limited with regard to validity and reliability: For example, this study used single items asking about difficulties in interacting with fellow students and lecturers. Therefore, tests for internal consistency could not be employed. These items could be operationalized differently, and the use of more sophisticated measures of social integration might yield different results.

### 4.2. Implications for Research and Practice

Given the fact that the findings are based on data from a single university, further research utilizing a broader, more representative database of students is recommended. Further investigation and evaluation studies are needed to validate the effects of counseling services, testing accommodations, and other interventions for students with depressive symptoms. In particular, mentoring programs have been shown to be a promising intervention to both improve social integration and prevent students from dropping out of university [80,81].

This study used established and validated objective instruments to measure academic performance and dropout intentions in order to generalize the results [46,82]. For instance, on-time graduation is one of the most commonly cited criteria for academic performance in Germany [82]. However, previous research suggests that the academic performance of university students is not only related to the completion of university studies or on-time graduation, but is also linked to academic and social experiences, personal growth, overcoming challenges, and maintaining a balance between studying and good health [83, 84, 85]. Consequently, it would be advisable to analyze the effects of the given predictors on the outcomes. Hence, an extended study period should not be viewed exclusively negatively and can also have a positive effect on academic performance in an individual context. Moreover, the decision to drop out of university can also be a judicious one under certain circumstances.

The survey exclusively documented the utilization of counseling services, excluding metrics pertaining to the extent of intervention and the number of sessions [21]. Further research is necessary to determine whether more intensive psychosocial support and extended therapy demonstrate significant longitudinal effects on the outcomes.

Overall, the results of this paper show that students are a vulnerable group with regard to mental health problems and underline the potential for prevention and health promotion in higher education [86]. As previously addressed by Hollederer [58], there is a need for structured student health management and the development of systematic student health monitoring to identify mental health problems, such as depression, in students at an early stage. This can help prevent their chronification, which has been shown to negatively affect academic performance and retention [26,28]. Despite the high demand for psychosocial counseling, it was found that the existing counseling services of the Studierendenwerk Kassel are not sufficiently used by students. However, the efficacy of intervention is dependent upon the timely identification and remediation of mental health problems. Therefore, it may be promising to better anchor the promotion of existing support services at the beginning of studies and also to inform staff in direct contact with students about these services. Finally, the existence of adequately equipped advice centers can be a factor that influences both the attractiveness of the university location and the general satisfaction of students with the framework conditions of their studies [21].

As demonstrated in the extant literature, social interaction with and support from fellow students is rated significantly worse by students with mental health problems compared to their peers [28,32]. However, it is an important resource that can alleviate academic challenges, positively influence mental health, and act as a protective factor against early dropouts, as previously reported [61,65]. To improve student-to-student collaboration in learning and working groups, universities can create opportunities for students to meet and learn together by creating specific formats, such as student mentoring programs. Positive experiences and interventions aimed at improving mental health literacy can increase the openness of other students and reduce prejudice towards students with mental health problems. Ultimately this has been shown to reduce the stigma of mental health problems among students [71]. Furthermore, by embedding inclusion goals, universities can consciously move towards an inclusive teaching and learning culture, creating an open and supportive climate for students with mental health issues [32].

## 5. Conclusions

Based on longitudinal data from a student survey at the University of Kassel, this analysis contributes to the existing literature on academic performance and dropout intentions among university students with mental health problems. The findings emphasize the significance of mental well-being in promoting academic success and mitigating the risk of dropout from higher education. This study highlights the need for a networked approach at universities, involving students, faculty, counseling services, and health management. Improving students’ mental health is a primary objective, with consideration given to the significance of social interactions in facilitating integration and psychosocial well-being. Concurrently, the objective is to destigmatize mental health issues, such as depression, with the aim of encouraging more students to utilize available support services. These services can assist students in managing distress, overcoming academic and personal challenges, and reducing the risk of dropping out of higher education.

## Figures and Tables

**Table 1:** Description of predictor, outcome variables, and covariates with means, standard deviations, and Cronbach’s α.

Variables	Description	Values	Mean (Standard Deviation) or Proportion in %		Cronbach’s *α*
			T0	T1	
Predictor Variables					
Depression Severity (PHQ-9 (sum variable)	Over the last 2 weeks, how often have you been bothered by any of the following problems? 1. Little interest or pleasure in doing things. 2. Feeling down, depressed, or hopeless. 3. Trouble falling or staying asleep, or sleeping too much. 4. Feeling tired or having little energy. 5. Poor appetite or overeating. 6. Feeling bad about yourself or that you are a failure or have let yourself or your family down. 7. Trouble concentrating on things, such as reading the newspaper or watching television. 8. Moving or speaking so slowly that other people could have noticed. Or the opposite being so fidgety or restless that you have been moving around a lot more than usual. 9. Thoughts that you would be better off dead or of hurting yourself in some way.	0 = not at all 1 = several days 2 = more than half the days 3 = nearly every day	7.71 (4.94) 31.2% ≥ 10 (MDD) 68.8% < 10 (Non-MDD)	8.68 (5.31) 32.6% ≥ 10 (MDD) 67.4% < 10 (Non-MDD)	0.86
Use of psychosocial counseling	Which of the following counseling or information services at the University of Kassel do you know and which have you used so far? Psychological advice Social advice	1 = used 0 = not used	Used = 8.4% Used = 4.6%	Used = 13.6% Used = 6.5%	n. a.* n. a.*
Academic performance					
Examination grades	What was the average grade (1.0 to 5.0) for your examination performance?	1.0 = very good 2.0 = good 3.0 = satisfactory 4.0 = fair 5.0 = poor	2.19 (0.70)	2.12 (0.61)	n. a.*
Deviation from the standard study period	Difference between the standard study period and individual study time (How many semesters is the standard study period in your current degree program? And in how many semesters do you expect to complete your current degree program, including the examination semester?)	−11.0–6.0 (range)	−1.69 (2.23)	−2.02 (2.37)	n. a.*
Social integration					
Difficulties with interaction with fellow students	Students experience the study situation differently. What causes difficulties for you personally? Difficulties for me are… Finding contacts with fellow students	1 = none 2 = few 3 = several 4 = strong	2.51 (0.97) None or few = 51.0% Several or strong = 49.0%	2.31 (1.01) None or few = 56.6% Several or strong = 43.4%	n. a.*
Difficulties with interaction with lecturers	Students experience the study situation differently. What causes difficulties for you personally? Difficulties for me are… The interaction with lecturers	1 = none 2 = few 3 = several 4 = strong	2.0 (0.83) None or few = 75.6% Several or strong = 24.4%	1.91 (0.79) None or few = 79.5% Several or strong = 20.5%	n. a.*
Outcome Variables					
Deviation from the standard study period	see predictor variables	−11.0–6.0 (range)	−1.69 (2.23)	−2.02 (2.37)	n. a.*
Dropout intentions (mean variable)	How much do the following statements apply to you? Currently, … I have decided to discontinue my studies altogether. … it is clear to me that I will discontinue my studies.	1 = not at all 2 = hardly 3 = partly 4 = rather yes 5 = yes	1.74 (0.86)	1.84 (0.99)	0.75
Covariates					
Marks of entrance certificate	What average grade did you have on the diploma that qualifies you to enter a degree program?	1.0 = very good 2.0 = good 3.0 = satisfactory 4.0 = fair 5.0 = poor	2.25 (0.62)	2.19 (0.60)	n. a.*
Financial burden	Until the end of your studies, you will only be able to earn your own money and support yourself to a limited extent. How much of a financial burden will this place on you and your family until the end of your studies?	1 = not at all 2 = hardly 3 = a little 4 = quite a lot 5 = very much	2.95 (1.14)	2.90 (1.11)	n. a.*

n. a.* = confirmational factor analysis was not applicable due to use of single items.

**Table 2:** Sociodemographic characteristics of the longitudinal sample.

Characteristics	Students Surveyed	
	n ^a^	Proportion
Total	500	100.0%
Age		
≤21 years	61	12.2%
21 ≤ 30 years	375	75.2%
≥30 years	63	12.6%
Female	326	65.2%
Male	166	33.2%
Foreign nationality	36	7.2%
First semester	148	29.6%
Intended degree		
Teacher education programs	75	15.0%
Bachelor	268	53.6%
Master	140	28.0%
Others (arts, diploma, others)	17	3.4%

^a^ n = sample size.

**Table 3:** Correlation matrix of predictor variables and outcome variables.

	Variables	1	2	3	4	5	6	7	8	9	10	11	12	13	14	15
1.	Severity of depression (PHQ-9) T0	-														
2.	Psychological advice T0 (1 = used)	0.16 **	-													
3.	Social advice T0 (1 = used)	0.13 **	0.15 **	-												
4.	Examination grades T0	0.16 **	−0.05	0.11 *	-											
5.	Difficulties interaction students T0	0.30 **	0.07	0.09 *	0.15 **	-										
6.	Difficulties interaction lecturers T0	0.28 **	0.09 *	0.14 **	0.19 **	0.35 **	-									
7.	Degree T0 (1 = bachelor’s)	0.13 **	−0.02	0.01	0.39 **	0.15 **	0.12 **	-								
8.	Grade of entrance certificate T0	0.12 **	−0.04	0.02	0.34 **	0.07	0.19 **	0.09 *	-							
9.	Financial burden T0	0.24 **	0.00	0.17 **	0.14 **	0.16 **	0.20 **	0.02	0.16 **	-						
10.	Gender T0 (1 = female)	0.12 **	0.03	−0.07	−0.04	−0.01	0.04	−0.04	−0.07 *	0.03	-					
11.	Age T0	−0.04	0.10 *	0.07 *	−0.05	−0.07	−0.07	−0.30 **	0.17 **	0.16 **	−0.13 **	-				
12.	Deviation from standard study period T0	−0.18 **	−0.15 **	−0.17 **	−0.25 **	−0.15 **	−0.14 **	−0.08	−0.18 **	−0.15 **	0.01	−0.15 **	-			
13.	Deviation from standard study period T1	−0.19 **	−0.19 **	−0.12 **	−0.15 **	−0.14 **	−0.13 **	−0.05	−0.18 **	−0.11 **	0.06	−0.16 **	0.66 **	-		
14.	Dropout intentions T0	0.32 **	0.07	0.08	0.33 **	0.28 **	0.32 *	0.06	0.17 **	0.10 **	−0.01	0.00	−0.20 **	−0.17 **	-	
15.	Dropout intentions T1	0.34 **	0.10 *	0.01	0.27 **	0.28 **	0.20 *	0.10 *	0.13 **	0.12 **	0.03	0.00	−0.22 **	−0.30 **	0.51 **	-
	n ^a^	499	497	497	366	500	500	500	498	498	492	499	498	500	499	458

Reported correlation coefficients are Spearman’s rank correlation coefficients; * *p* < 0.05, ** *p* <0.01. A *p*-value of less than 0.05 is considered statistically significant, significant correlation coefficients on outcomes in bold; for variable descriptions, see “Materials and Methods”; ^a^ sample size.

**Table 4:** Standardized regression coefficients of OLS linear regression analysis for the predictive values of PHQ-9, psychosocial counseling, and covariates for the deviation from the standard study period.

	Deviation from the Standard Study Period T1 (n ^a^ = 483)				
Predictor Variables (Baseline)	M1	M2	M3	M4	M5
	β ^b^	β	β	β	β
Baseline control of outcome ^c^	0.706 ***	0.702 ***	0.690 ***	0.680 ***	0.662 ***
Gender (1 = female)		0.060	0.070 *	0.069 *	0.068 *
Age		−0.033	−0.043	−0.040	−0.040
Depression severity (PHQ-9)			−0.082 *	−0.072 *	−0.067
Psychosocial counseling					
Psychological advice (1 = used)				−0.048	−0.055
Social advice (1 = used)				−0.030	−0.030
Covariates					
Grades of entrance certificate					−0.055
Financial burden					−0.003
**R^2^** ^d^	**0.499**	**0.504**	**0.510**	**0.514**	**0.518**
**R^2^adj** ^d^	**0.498**	**0.501**	**0.506**	**0.507**	**0.509**

Significance levels: * < 0.05; ** < 0.01; *** < 0.001. A *p*-value of less than 0.05 is considered statistically significant; for variable descriptions, see “Materials and Methods”; ^a^ sample size; ^b^ standardized regression coefficients; ^c^ baseline control of outcome: deviation from the standard study period at T0; ^d^ R^2^—coefficient of determination; adjusted R^2^—adjusted coefficient of determination.

**Table 5:** Standardized regression coefficients of OLS linear regression analysis for the predictive values of PHQ-9, academic performance, and social integration for dropout intentions.

	Dropout Intentions T1 (n = 332) ^a^					
Predictor Variables (Baseline)	M1	M2	M3	M4	M5	M6
	β ^b^	β	β	β	β	β
Baseline control of outcome ^c^	0.515 ***	0.515 ***	0.457 ***	0.413 ***	0.401 ***	0.406 ***
Gender (1 = female)		0.015	−0.007	−0.006	−0.007	−0.007
Age		−0.006	0.004	−0.007	−0.003	−0.002
Depression severity (PHQ-9)			0.204 ***	0.190 ***	0.169 ***	0.163 **
Academic performance						
Deviation from standard study period				−0.073	−0.060	−0.056
Examination grades				0.108 *	0.106 *	0.098
Social integration						
Difficulties with interaction students					0.099	0.095
Difficulties with interaction lecturers					−0.021	−0.029
Covariates						
Degree (1 = Bachelor)						0.014
Financial burden						0.033
**R^2^** ^d^	**0.265**	**0.266**	**0.303**	**0.321**	**0.329**	**0.330**
**R^2^adj** ^d^	**0.263**	**0.259**	**0.295**	**0.308**	**0.312**	**0.309**

Significance levels: * < 0.05; ** < 0.01; *** < 0.001. A *p*-value of less than 0.05 is considered statistically significant; for variable descriptions, see “Materials and Methods”; ^a^ n sample size; due to missing values, sample size ranged from 332 to 458; ^b^ standardized regression coefficients; ^c^ baseline control of outcome: dropout intentions at T0; ^d^ R^2^—coefficient of determination; adjusted R^2^—adjusted coefficient of determination.

## Data Availability

The data presented in this study are available on request from the corresponding author. The data are not publicly available due to the privacy of the participants and ethical concerns.
